# Patients’ primary activities prior to critical illness: how well do clinicians know them and how likely are patients to return to them?

**DOI:** 10.1186/s13054-018-2283-7

**Published:** 2018-12-17

**Authors:** Alexi T. Gosset, Michael C. Sklar, Aaron M. Delman, Michael E. Detsky

**Affiliations:** 1 Harvard University, Boston, Massachusetts USA; 2 Department of Medicine, Sinai Health System, Toronto, Ontario Canada; 3 Interdepartmental Division of Critical Care Medicine, University of Toronto, 600 University Ave, Suite 18-232-1, Toronto, ON M5G 1X5 Canada; 4 Department of Surgery, University of Cincinnati School of Medicine, Cincinnati, Ohio USA; 5 Palliative and Advanced Illness Research (PAIR) Center, University of Pennsylvania Perelman School of Medicine, Philadelphia, Pennsylvania USA

Admission to the intensive care unit (ICU) can make patients feel anonymous and depersonalized [1]. Knowledge of a patient’s primary activity can mitigate the risk of depersonalization by providing insight into a patient’s values, preferences, and overall function. A patient’s primary activity is defined by how they report spending their free time. This information can be used to engage in shared decision-making, ensuring patients receive care that is goal-concordant based on the feasibility of recovering from their critical illness [2]. Therefore, we conducted a prospective observational study to determine if ICU physicians and nurses could identify their patients’ primary activities. Other objectives included determining if patients were able to return to these activities and the probability of patients surviving based on their primary activity.

From October 2013 to May 2014 [3], enrolled patients (or their surrogates) were asked to identify their primary activity prior to hospitalization (Table 1). Attending physicians and nurses on admission days 3–6 were asked to identify this activity. Patients were followed to 6 months after enrollment to assess if they had survived and returned to their activities.

**Table 1:** Activity category and frequency, description, ability to return to activity, and survival

Activity category	Description and examples	Full return to activity (%)^a^	Did not fully return to activity (%)^b^	Deceased (%)^c^	Unknown (%)^d^	Total
Employment	Work, vocation, or employment status	33 (38)	26 (30)	28 (32)	1 (1)^e^	88
Student	Involves school or academics	2 (67)	1 (33)	0 (0)	0 (0)	3
Physical activity	Physical exercise or strain (i.e., weight lifting, walking)	13 (45)	4 (14)	12 (41)	0 (0)	29
Household	Chores requiring some amount of activity (i.e., cleaning house, shopping)	17 (32)	10 (19)	24 (45)	2 (4)	53
Active	Involves activity but not as main focus (i.e., traveling, fishing)	4 (44)	1 (11)	4 (44)	0 (0)^e^	9
Social	Engaging with other people (i.e., family time, visiting friends, therapy)	14 (45)	2 (6)	13 (42)	2 (6)	31
Active sedentary	No physical strain but requires active engagement (i.e., arts and crafts, reading)	7 (28)	1 (4)	15 (60)	2 (8)	25
Passive sedentary	No physical strain and no active engagement (i.e., watching TV)	20 (50)	0 (0)	17 (43)	3 (8)	40
Not reported	No activity listed	NA	NA	17 (68)	8 (32)	25
Total	NA	110 (36)	45 (15)	130 (43)	18 (6)	303

^a^Frequency and percentage of patients within each activity category that were alive and fully returned to their primary activity 6 months post-enrollment in the study. All percentages calculated by dividing the frequency by the activity type’s total

^b^Frequency and percentage of patients within each activity category that were alive but did not fully return to their primary activity 6 months post-enrollment in the study

^c^Frequency and percentage of patients within each activity category that were deceased 6 months post-enrollment in the study

^d^Frequency and percentage of patients within each activity category with unknown vital and/or return to pastime status 6 months post-enrollment in the study

^e^Percentages do not add to 100% due to decimal place rounding

We found that clinicians had low rates of reporting knowledge of their patients’ primary activities at 13% (38/303) and 12% (35/300) for nurses and physicians, respectively. Patients’ primary activities were reported correctly for 7% (20/303) and 5% (15/300) of patients by nurses and physicians, respectively (Table 1). Among patient reported activities, the most frequent were employment (29%, 88/303) and household work (17%, 53/303). Among survivors 64% (110/173) could perform their primary activity at 6 months, 26% (45/173) could not. For 10% (18/173) of survivors we were unable to confirm if they returned to their primary activity (Table 2).

**Table 2:** Physician and nurse accuracy in predicting patient primary activities

	Physicians (*n* = 300; %)^a^	Nurses (*n* = 303; %)^a^
Correct^b^	15 (5)	20 (7)
Incorrect^c^	18 (6)	13 (4)
No patient response^d^	2 (1)	5 (2)
No clinician response^e^	265 (88)	265 (87)

^a^Total number of responses and percentage relative to total patient count

^b^Instances where clinician and patient primary activity responses agreed

^c^Clinician and patient primary activity responses disagreed

^d^Patient provided no activity response but the clinician did

^e^Clinician failed to provide an activity response

We believe that knowing how patients spend their time prior to their illness can help in shared decision-making and ensure the delivery of goal-concordant care [4]. In our study, ICU clinicians rarely reported knowing their patient’s primary activity and were correct in only half of those responses, suggesting that ICU clinicians lack an understanding of their patients’ lives prior to critical illness. This is consistent with previous work that assessed physicians’ knowledge of patients’ broader values [5]. The systematic collection of information related to patients’ values may mitigate the risk of depersonalization. Further work is needed to understand the potential impact of whether knowledge of patient activities leads to improved health outcomes and the delivery of goal-concordant care.

## Abbreviations

ICU Intensive care unit
